# Does free schooling affect pathways from adverse childhood experiences via mental health distress to HIV risk among adolescent girls in South Africa: a longitudinal moderated pathway model

**DOI:** 10.1002/jia2.25262

**Published:** 2019-03-14

**Authors:** Franziska Meinck, FM Orkin, Lucie Cluver

**Affiliations:** ^1^ Centre for Evidence‐Based Intervention Department of Social Policy and Intervention University of Oxford Oxford United Kingdom; ^2^ OPTENTIA Faculty of Health Sciences North‐West University Vanderbijlpark South Africa; ^3^ MRC Developmental Pathways for Health Research Unit School of Clinical Medicine University of the Witwatersrand Johannesburg South Africa; ^4^ Department of Psychiatry and Mental Health University of Cape Town Cape Town South Africa

**Keywords:** sexual risk behaviour, adolescent girls, mental health, adverse childhood experiences, social protection

## Abstract

**Introduction:**

Adolescent girls are at high risk of HIV infection in sub‐Saharan Africa. Mental health distress, driven by adverse childhood experiences (ACEs) such as abuse, poverty and family HIV, may be an important driver of HIV risk behaviour among adolescent girls, while education may mitigate these risks. This study aimed to develop an empirically based theoretical model between ACEs, mental health distress and HIV risk behaviour among adolescent girls in South Africa and to investigate the potential moderating effects of free schooling provision.

**Methods:**

Self‐report questionnaires using validated scales were completed by adolescent girls aged 12 to 17 at baseline in two provinces in South Africa in 2011, with a 99% one‐year follow‐up in 2012 (n = 1498). Sampling included every household in randomly selected census enumeration areas of four deprived health districts. Confirmatory factor analysis was employed to identify measurement models and a structural equation model was developed to test pathways of risk and protection.

**Results:**

Internalizing and externalizing mental health distress fully mediated the positive relationship between ACEs at baseline and HIV risk behaviour at follow‐up among adolescent girls. Internalizing mental health distress was associated with increased sexual risk at follow‐up via higher externalizing problems. Free schooling provision at baseline and follow‐up eliminated the pathway from internalizing to externalizing mental health distress by moderating the pathway between ACEs and internalizing mental health distress. It also weakened the pathway from externalizing mental health distress to HIV risk behaviour at follow‐up through a direct negative effect on externalizing mental health distress.

**Conclusions:**

Reducing ACEs and adolescent mental health distress is essential for reducing HIV risk behaviour among girls in South Africa. Free schooling provision may be an important tool for reducing these problems and mitigating negative pathways to HIV risk among vulnerable adolescent girls.

## 1 Introduction

Human immunodeficiency virus (HIV) remains a major public health concern in sub‐Saharan Africa. Despite impressive global achievements in access to treatment and declines in overall AIDS‐related mortality and morbidity, AIDS remains the leading cause of death among adolescents in Africa 1. New infections are unequally distributed among genders, with young women 44% more affected than young men 2.

There is increasing evidence that adolescent HIV risk behaviours are associated with social, psychological and economic vulnerability. Previous studies in the US showed clear cumulative associations between adverse childhood experiences (ACEs) such as physical, emotional and sexual abuse, exposure to domestic violence, or parental absence and increased risk for HIV risk behaviour among women 3. A recent global meta‐analysis found a combined odds ratio of 5.92 for exposure to four or more ACEs and sexually transmitted diseases in adulthood 4. In sub‐Saharan Africa, factors associated with increased HIV risk behaviour include ACEs such as being AIDS‐affected, poverty, abuse and psychological distress 5, 6. However, actual pathways between ACEs and HIV risk behaviours are not well understood.

A potential pathway by which ACEs may increase HIV risk behaviours is through mental health distress. It is well established that exposure to ACEs is associated with an increased risk for depression, suicide ideation, anxiety, delinquency and behaviour problems 4, with internalizing mental health distress preceding externalizing distress 7. Associations between poor mental health and HIV risk behaviour have been shown in multiple studies in sub‐Saharan Africa 8, 9, 10, however, the role of mental health in pathways from ACEs to HIV risk behaviours is under‐examined.

In recent years, there has been an increasing interest in the association between education and HIV risk behaviour as a potential mechanism for intervention, particularly among adolescent girls. For children in low‐resource settings and affected by HIV, paying school fees can be a substantial strain on households, causing school dropout and uncertainty regarding education. Qualitative work identifies that this is a source of distress for children 11. Recent research evidence suggests that school non‐attendance is linked to higher HIV risk behaviour in multiple contexts 12, 13 while school attendance and increases in education levels have been shown to significantly reduce HIV acquisition and prevalence 14, 15, 16, 17. Recent randomized controlled trials (RCTs) show that free‐schooling social protection, including school feeding, school uniform and tuition fees, decreases rates of school‐dropout and HIV risk behaviour among orphaned children 18, 19, 20. Furthermore, experimental and quasi‐experimental studies have shown that “cash,” “care” and “classroom” (free schooling) social protection interventions in combination or by themselves can be effective in reducing HIV risk among adolescent girls 21, 22, 23. A previous study has shown lower HIV risk behaviour and suicidality among adolescents with high school connectedness 24 but thus far no study examines whether classroom interventions reduce adolescent mental health distress. Questions also remain about whether these social protection interventions, and in particular free schooling, work by affecting the pathways to HIV risk behaviour among adolescent girls in areas with high HIV prevalence. An understanding of such mechanisms is important to develop effective interventions. This study presents a unique opportunity to examine the hypothesized beneficial effects of the national free schooling roll‐out in South Africa on pathways to HIV risk behaviour among adolescent girls in HIV endemic settings.

**Figure 1:**
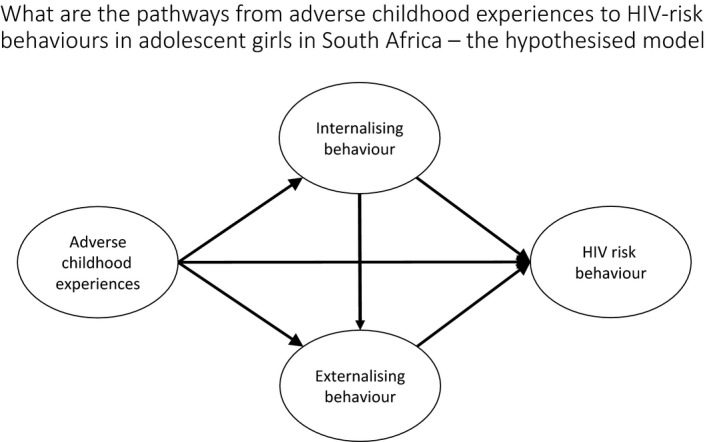
Hypothesized path model from adverse childhood experiences to HIV risk behaviour among adolescent girls.

The current paper therefore has two aims: (1) To develop a theoretical pathway model by investigating hypothesized associations from ACEs to HIV‐risk behaviours via internalizing and externalizing mental health distress in adolescent girls in South Africa (Figure 1). (2) To further develop intervention theory by investigating whether the established pathways between ACES, mental health distress and HIV risk behaviours obtain when moderated by classroom social protection in the form of free schooling.

## 2 Methods

### 2.1 Participants and procedure

Adolescents (n = 3516) aged 10 to 17 (56.7% female) were recruited from urban and rural areas in two provinces in South Africa and interviewed at baseline (2010 to 2011) and follow‐up (2011 to 2012). Retention rate at one‐year follow‐up was 96.8%, refusal rate at baseline was <2.5%. Adolescents were recruited through door‐to‐door sampling in randomly selected census enumeration areas in four health districts with antenatal HIV‐prevalence >30%. One randomly selected adolescent was interviewed per household, for 70 minutes, by interviewers trained in working with vulnerable youth. No exclusion criteria were applied unless the adolescent was deemed incapable of giving consent or understanding the questionnaire. The sample thus included children with chronic illnesses, physical disabilities and severe mental health problems. Informed consent was sought from both the primary caregiver and the adolescent. Consent forms and questionnaires were translated into Zulu, Xhosa, Sotho, Sepedi, Swati and Tsonga and checked with back translation.

Ethical clearance was provided by the Universities of Oxford (SSD/CUREC2/09‐52), Cape Town (389/209) and KwaZulu‐Natal (HSS/0254/09) and by the provincial government departments of Health and Education. Participants were interviewed in private spaces (i.e. gardens) to ensure privacy and confidentiality. Confidentiality was maintained throughout the study except where participants requested help or were at risk of significant harm. Then referrals were made to health or child protective services with follow‐up support. There were no monetary incentives, but all participants received a certificate and refreshments.

### 2.2 Measures

The *adverse childhood experiences latent construct* was measured as follows: Lifetime *child abuse* was measured using five physical and emotional abuse items from the UNICEF Scales for National‐Level Monitoring of Orphans and Other Vulnerable Children 25 and two sexual abuse items designed by local social workers. Items were summed to create a total child abuse score (α = 0.66). *Poverty* was measured using one item on the number of days of food insufficiency in the household in the past week. *Family AIDS* was measured using Verbal Autopsy methods validated in previous studies of adult mortality in South Africa 26. Determination of caregiver HIV/AIDS‐illness required three or more AIDS‐defining illnesses (i.e. tuberculosis, shingles or Kaposi's sarcoma) or reported HIV positive status. *Witnessing domestic violence* was measured using one item querying the number of days where adults were hitting each other in the household in the past week. The *internalizing mental health latent construct* was measured as follows: *Depression* in the past two weeks was measured using the Children's Depression Inventory which has been previously used in South Africa and has acceptable internal consistency in this sample (α = 0.73). *Anxiety* was measured using the Revised Children's Manifest Anxiety Scale 27 which has been validated in South Africa 28 and showed good internal consistency in this sample (α = 0.84). *Suicidal ideation* during the past month was measured using the Mini International Psychiatric Interview for Children and Adolescents (Mini‐Kid) 29 which has been previously used in South Africa and showed acceptable internal consistency in this sample (α = 0.73). The *externalizing mental health latent construct* was measured using the following: *Alcohol and drug use* in the past month was measured using two items from the National Survey of HIV and Risk Behaviors amongst Young South Africans 30. *Behaviour problems* in the past six months were measured using the delinquency subscale of the Child Behaviour Checklist (α = 0.58) 31. *Peer relationship problems* in the past six months were measured using the Peer Problems subscale of the Strengths and Difficulties Questionnaire (α = 0.60) 32. All have been previously used in South Africa. The *HIV risk behaviour latent construct* was measured using three binary items from the National Survey of HIV and Sexual Behavior among Young South Africans 30 measuring past year *infrequent condom use,* lifetime *sex whilst drunk or on drugs* and *multiple partners* (>2 in the past year). *Free schooling social protection* was measured using three items on whether or not children went to a *no‐fees school*, received a *free school meal* and *free text books* and defined as receiving all three provisions at baseline and follow‐up versus not receiving all three at baseline and/or follow‐up*. Pre‐selected covariates urban/rural location* and *age* were also collected.

### 2.3 Analysis

Analyses were conducted in four stages with Stata 14.2 and MPlus 8 using the longitudinal sample of adolescent girls (n = 1919). Few adolescents younger than 12 years reported being sexually active (n = 9) and thus analyses were limited to the sample of adolescent girls aged 12 to 17 years at baseline (retention 99.3%; n = 1498 at follow‐up). There were no differences between those retained and lost to follow‐up at baseline. First, descriptive statistics for all outcomes, potential mediators, school social protection variables and covariates were calculated (Table 1). Second, measurement models were assessed for all latent constructs, which comprised sum scores, dichotomous variables or both. Third, latent constructs were included in a model with hypothesized pathways from ACEs via adolescent mental health to HIV‐risk behaviour, controlling for baseline HIV risk behaviour, age and urban/rural location. Non‐significant pathways were dropped and small modifications made to improve model fit. These modifications included re‐specification of covariance between error terms 33. Finally, hypothesized moderation of pathways through the free schooling variable was tested in three sequential models, each testing the moderation on a separate pathway of the model 34. Analyses were conducted in MPlus 8, using weighted least squares and variance adjusted estimation due to categorical data of the outcome construct indicators. As some variables were non‐normally distributed, all parameters were also estimated using the bootstrapping procedure with 1000 bootstrapped samples. Model fit was assessed using χ^2^/d.f., comparative fit index (CFI), root mean square error of approximation (RMSEA), standard root mean residual (SRMR) and weighted root mean square residual (WRMR). By convention, the maximum acceptable value for χ^2^/d.f. is 5. For CFI, a value >0.95 indicates good fit and >0.90 adequate fit. For RMSEA and SRMR a value of 0.05 indicates good model fit, and for WRMR a value close to 1 indicates good model fit. Bayesian Information Criterion (BIC) and Akaike Information Criterion (AIC) were applied for testing the model fit improvement of moderation against the specified baseline model as other model fit criteria were not available for interactions of latent variables in MPlus. For AIC and BIC smaller values indicate better model fit 35. Missing data were less than 1% on all variables and thus no imputation was conducted.

**Table 1:** Socio‐demographic characteristics at baseline and follow‐up among vulnerable South African girls (n = 1498)

	Baseline % (n)	Follow‐up % (n)
Mean age	14.3 (SE 0.043) SD 1.66	15.54 (SE 0.05) SD 1.78
Rural Area	52.1 (781)	52.3 (784)
AIDS‐ill caregiver	34.3 (514)	19.3 (289)
Poverty (days without food/week, Scale 0 to 7)	0.94 (SE 0.04) SD 1.59	0.98 (SE 0.04) SD 1.63
Any abuse (lifetime)	54.2 (812)	65.4 (979)
Past week domestic violence (past week)	6.6 (99)	5.3 (79)
Anxiety (Scale 0 to 14)	5.02 (SE 0.09) SD 3.55	3.99 (SE 0.09) SD 3.33
Suicidal ideation (past month, Scale 0 to 5)	0.49 (SE 0.03) SD 1.09	0.49 (SE 0.03) SD 1.11
Depression (past two weeks, Scale 0 to 16)	2.16 (SE 0.07) SD 2.72	1.50 (SE 0.06) SD 2.18
Delinquency (past six months, Scale 0 to 14)	1.86 (SE 0.05) SD 2.12	2.03 (SE 0.05) SD 2.13
Alcohol and drug use (past month)	6.8 (102)	25.2 (377)
Peer problems (past six months, Scale 0 to 18)	4.17 (SE 0.09) SD 3.30	4.02 (SE 0.08) SD 3.21
Inconsistent condom use (past year)	8.5 (127)	11.7 (176)
Multiple sex partners (>2) (past year)	4.3 (64)	8.0 (120)
Sex drunk or on drugs (ever)	1.4 (21)	2.4 (36)
Free schooling (receipt at both time points)	71.0 (1063)	

## 3 Results

At baseline adolescent girls had a mean age of 14.3 years (range 12 to 17). 34.3% had a family member ill with AIDS. They reported a mean of 1.9 days without sufficient food in the household and 2.9 abusive events in their lifetime. Mean scores for depression, anxiety, suicidal ideation, peer problems, alcohol and drug use and delinquency can be found in Table 1. At follow‐up 11.7% reported inconsistent condom use, 2.4% reported having sex drunk or on drugs and 8.0% had multiple sexual partners over the past year. 71.0% of participants received free schooling provision (Table 1). Sample proportions of adolescent girls’ risk exposure by HIV‐risk category can be found in Table S1. Proportions and means for the free schooling provision by different risk exposures can be found in Table S2 and show no systematic differences.

### 3.1 Measurement models

Confirmatory factor analysis was exploited to examine hypothesized latent constructs for the predictors, mediators and outcome. A simultaneous confirmatory factor analysis was carried out to examine model fit of the individual constructs (Table S3). The ACEs latent construct was identified by the total score for the poverty measure, the total score for child abuse victimization, and familial AIDS at baseline, which all loaded >0.4. Domestic Violence was removed from the construct as its factor loading was <0.2. The indicators for the latent construct of internalizing mental health distress were the respective averages of depression, anxiety and suicide ideation sum scores at baseline and follow‐up 36. They loaded onto the construct >0.4. The latent construct for externalizing mental health distress was identified by the respective averages of child delinquency, peer problems, and drug and alcohol abuse sum scores at baseline and follow‐up 36. They loaded onto the construct >0.3. The HIV risk behaviour latent construct was identified by the dichotomous variables of frequent condom use, sex drunk or on drugs, and having multiple sexual partners at follow‐up, with all indicators loading onto the construct >0.6. All indicator variables within each of the four constructs had equal weight (Table S3). Model fit for the simultaneous CFA was good on all criteria except CFI, which was adequate (χ^2^ = 223.23, d.f. = 48, *p *<* *0.001, χ^2^/d.f. = 4.65, CFI 0.916, WRMR 1.428, RMSEA 0.049 (0.043 to 0.056)).

### 3.2 Path model

**Figure 2:**
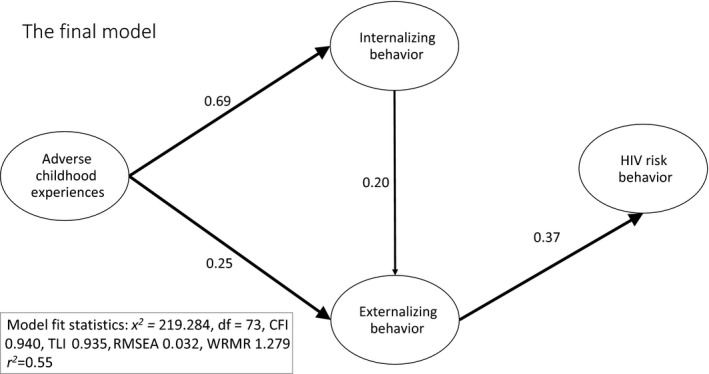
Empirical pathway model from adverse childhood experiences to HIV risk behaviour among adolescent girls.

The four latent constructs were therefore included in the hypothesized model with the following pathways (Figure 1): ACEs associated with internalizing and externalizing mental health distress and HIV risk behaviour, internalizing mental health distress associated with externalizing mental health distress and each associated with HIV risk behaviour (Table 2). The hypothesized model had poor model fit (χ^2^ = 342.931, d.f. = 72, *p *<* *0.001, χ^2^/d.f. = 4.76, CFI 0.889, RMSEA 0.050, WRMR 1.561). All non‐significant pathways were then removed from the model one pathway at a time: non‐significant covariates, the direct effect of ACES on HIV risk behaviour, and the direct effect of internalizing mental health distress on HIV risk behaviour. This resulted in the following adequate model fit: (χ^2^ = 352.117, d.f. = 77, *p *<* *0.001, χ^2^/d.f. = 4.57, CFI 0.900, RMSEA 0.049, WRMR 1.622). Further modifications were carried out based on modification indices as specified in Table S4. The final model shows direct paths from ACEs to internalizing (β = 0.69, *p *<* *0.001) and externalizing mental health distress (β = 0.25, *p *=* *0.005), from internalizing to externalizing mental health distress (β = 0.20, *p *=* *0.028) and from externalizing mental health distress to HIV‐risk behaviour (β = 0.37, *p *<* *0.001) (Figure 2).

**Table 2:** Mediation model from ACEs to HIV risk behaviour among adolescent girls (n = 1498)

Predictor	Adverse experiences	Internalizing	Externalizing	Sexual Risk
Adverse experiences	–	0.689***	0.251*	ns
Internalizing	–	–	0.196*	ns
Externalizing	–	–	–	0.370***
Age	0.100*	0.128***	0.075*	0.361***
Baseline sexual risk	0.141***	0.068*	0.207***	0.291***
Urban location	ns	ns	0.064*	ns
Correlated item residuals	Peer problems with depression: 0.245***; Alcohol with delinquency: −0.429***			
Goodness of fit	*χ* ^2^ (d.f.): 2552.965 (73), CFI 0.940; TFI 0.917, RMSEA 0.037 (0.031 to 0.042),			
r^2^	0.039	0.544	0.288	0.552

– Refers to non‐applicable pathways, ns refers to pathways that were removed in the mediation model. ACEs, adverse childhood experiences; CFI, comparative fit index; RMSEA, root mean square error of approximation.

All effects reported are standardized, ***p <* *0.001, *p <* *0.05.

Three indirect effects were established: (1) from ACEs via internalizing to externalizing mental health distress (β = 0.14, *p *=* *0.023); (2) from adverse experiences via externalizing mental health distress to HIV risk behaviour (β = 0.09, *p *=* *0.011); and (3) from internalizing via externalizing mental health distress to HIV risk behaviour (β = 0.07, *p *=* *0.046, Figure 2). The final model accounted for 55% of the variance in HIV risk behaviour. Model fit was good on all criteria set out above except CFI, which was adequate: (χ^2^ = 219.284, d.f. = 73, *p *<* *0.001, χ^2^/d.f. = 3.00, CFI 0.940, RMSEA 0.032, WRMR 1.279). AIC (58654.4) and BIC (58888.12) were also obtained for this model to allow comparison with the final model.

**Figure 3:**
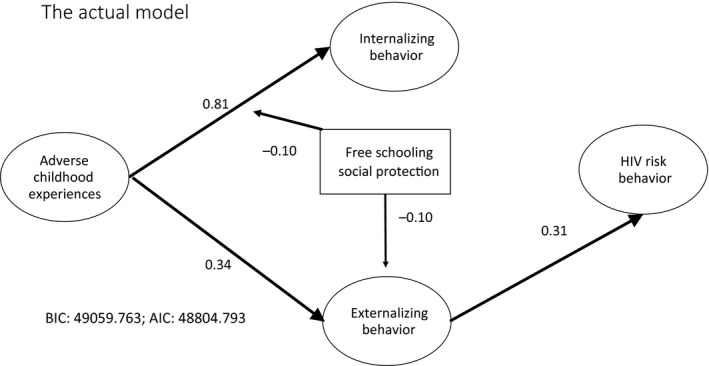
Final model with moderation: direct effect and moderating effect of free schooling social protection on pathways from adverse childhood experiences to HIV‐risk behaviour among adolescent girls.

Direct effects of free schooling provision on the latent constructs, as well as interactions between free schooling social protection and the latent constructs, were examined one at a time because of the computationally heavy demands of categorical latent constructs [37, *p*. 471]. AIC and BIC were examined in each case and compared to the mediation model in Figure 2. In the first instance, no significant direct effect of free schooling provision on internalizing mental health distress was found (Table S5, Model 1). There was a significant interaction between free schooling provision and ACEs (β=−0.11, *p *=* *0.008) moderating the pathway from ACEs to internalizing mental health distress. A direct effect of free schooling provision was found on externalizing mental health distress, but no interaction effect between ACEs and free schooling provision was found to moderate the pathway between ACEs and externalizing mental health distress. Furthermore, the introduction of free schooling provision renders the pathway from internalizing to externalizing mental health distress insignificant. In the second instance, a significant direct effect of free schooling provision could be observed on externalizing mental health distress (β=−0.10, *p *=* *0.008), but no interaction effect between internalizing behaviour and free schooling social protection was found (see Table S5, Model 2). In the third instance, neither a direct effect of free schooling social protection on HIV risk behaviour nor an interaction effect between free schooling social protection and externalizing mental health distress was found (see Table S5, Model 3). A final model was then run which included the significant interaction effect and the direct effect of free schooling provision as well as the significant mediation pathway (Figure 3, Table 3).

**Table 3:** Final model including all significant mediation pathways and the significant direct and interaction effects of free schooling social protection on HIV risk among South African adolescent girls (N = 1498)

	Final model			
	ACEs	Internalizing	Externalizing	Sexual risk
ACEs	–	0.808***	0.344***	ns
Internalizing	–	–	ns	ns
Externalizing	–	–	–	0.306
Free schooling social protection	–	ns	−0.092*	ns
ACE × free schooling	–	−0.099*	–	–
Internalizing × free schooling	–	–	ns	–
Externalizing × free schooling	–	–	ns	–
Sexual risk × free schooling	–	–	–	ns
Age	0.100*	0.125***	0.086**	0.448***
Baseline sexual risk	0.145**	ns	0.184***	0.189***
Rural location	ns	ns	0.094**	ns
Measurement models				
AIDS‐ill caregiver	0.295***			
Child abuse	0.606***			
Poverty	0.349***			
Suicide ideation		0.635***		
Anxiety		0.743***		
Depression		0.664***		
Peer problems			0.191***	
Delinquency			0.748***	
Drug and alcohol use			0.580***	
Inconsistent condom use				0.802***
Multiple partners				0.814***
Sex drunk or on drugs				0.721***
Goodness of fit	BIC: 49059.763; AIC: 48804.793			

– Refers to non‐applicable pathways, ns refers to non‐significant pathways that were removed in the mediation model. AIC, Akaike Information Criterion; BIC, Bayesian Information Criterion.

All effects reported are standardized, ***p <* *0.001, **p <* *0.005, *p <* *0.05.

## 4 Discussion

Reducing HIV risk among adolescent girls is a huge challenge, particularly in HIV‐endemic countries. The empirical model tested in this study shows a set of pathways of vulnerability leading to increased HIV risk behaviour. It is noteworthy that no significant direct pathway was found between ACEs and HIV risk behaviour. The relationship between ACEs and HIV risk behaviour was fully mediated by internalizing and externalizing mental health distress. Moreover, there was no significant pathway from internalizing mental health distress to HIV risk behaviour. This supports the conception of internalizing symptoms preceding externalizing symptoms 7. Earlier studies, which tested correlations and not pathways found strong associations between ACEs and HIV risk behaviour as well as between internalizing and HIV risk behaviour 38, 39, 40. However, these did not test theoretical pathway models for HIV risk behaviour and therefore had not tested for mediation. This study demonstrates pathways through mental health distress and thereby provides new evidence for opportunities to interrupt cycles of increased risk.

This study also provided evidence that classroom social protection in the form of free schooling for adolescents (i.e. free schooling, school‐books and school meals) is associated with lower externalizing mental health distress and modifies the risk pathway from ACEs to internalizing mental health distress. This could operate through a number of mechanisms which need to be further explored: for instance, adolescent girls who receive free schooling may experience lower mental health distress because they do not have the additional worry of being able to pay the school fees 11. In addition, by receiving free schooling social protection, adolescent girls receive one hot meal a day which may prevent hunger and thus reduce risk for mental health distress 41. Further school social protection may increase school attendance 42, giving girls the chance to make friendships and build support networks. This study suggests that free education is an important tool to promote mental health among adolescent girls in South Africa, and thereby mitigate pathways to HIV risk behaviour. However, HIV risk behaviour and mental health distress vary across age group and thus may require age‐sensitive interventions. Therefore, further research could valuably investigate which age groups benefit most from classroom social protection for the prevention of HIV risk behaviours. Future qualitative research should also examine why free schooling is directly associated with reducing externalizing mental health distress and also modifies the risk pathway to internalizing mental health distress.

This study has notable limitations. First, data are available only from two time points, and although mean factor scores of baseline and follow‐up mental health data were used for the mediator variables to establish temporal sequence, causality cannot be asserted. While reverse causality might be unlikely for many of the pathways, for some pathways – particularly between internalizing and externalizing mental health distress – bi‐directionality is possible. However, the cautious attribution of causal order with temporal precedence, and based on plausible theoretical grounds, is well established in structural equation modelling 33. Future research using rigorous randomized designs is needed to examine the effectiveness of free‐schooling social protection on HIV risk behaviour among adolescent girls. Such studies can complement the existing evidence on orphaned children only 19 and of cash social protection on HIV risk behaviour 14 and school attendance 43.

Second, data were collected in 2011 and 2012 and results and potential intervention requirements need to be interpreted with this in mind. Non‐fee schools and the school feeding programme were started in 2006/2007 with schools in lowest income areas and have since been extended across the country. They reach nine million children in schools in deprived areas and thus effects of these programmes may be different now that they have been scaled up 44. However, testing intervention effects at the start of rollout when an intervention is not universally available does allow determination of differences between those that are in receipt and not in receipt of it and thus this can be considered a real strength of the presented data. Third, receipt of social protection was only measured at baseline and follow‐up and it is therefore impossible to distinguish between children who have been in long‐term receipt of the intervention or those who are recent recipients. Fourth, all items were measured using adolescent self‐report only. As child abuse and HIV risk behaviours are highly stigmatized, some underreporting may have occurred. However, other types of respondents, that is, caregivers have been shown to be even more unreliable 45 and thus adolescent self‐report is preferred over caregiver report. Fifth, HIV risk taking behaviours are low in this sample, possibly due to the young age of the participants (mean age 15.5 years at follow‐up), and this will have implications for programming. Sixth, this study did not use randomization for the intervention and thus some selection bias cannot be ruled out as students receiving the interventions were in the most highly deprived areas of South Africa and thus may have experienced higher levels of poverty, mental health distress and adversity. Seventh, this study focused on pathways which increase HIV risk behaviour between latent constructs, as moderated by free schooling provision. Future studies should examine pathways of additional resilience, protective factors and the contributions of each of the exposures towards HIV risk behaviour. Eighth, this study focused on adolescent girls, as the group within the region at highest risk of HIV infection. Future research should investigate pathways to HIV‐risk behaviours among boys and examine the effects of free schooling and other potential moderators on these pathways. Finally, the study only sampled adolescents in highly deprived areas of South Africa and is thus not representative of the overall population. However, the study benefited from in‐sample variation that included participants from six different language groups, and is therefore likely to be indicative for vulnerable adolescent girls in deprived areas in South Africa.

This study also has multiple strengths. It is the first longitudinal study to test multiple pathways associated with increased HIV risk behaviour among girls in South Africa. It moves the analysis beyond direct associations by indicating the moderating effect of a feasible intervention, free schooling. The sample size was large, participation high, attrition at follow‐up low and model fit good.

## 5 Conclusions

Free access to education, school feeding and books at primary and secondary school level may be an important part of an HIV prevention package for adolescent girls in South Africa. Previous research has focused on single provisions such as grants to alleviate poverty, access to education to reduce risk behaviour, or parenting to reduce abuse and improve adolescent mental health 14, 22, 46. In recent years, research focus has shifted to combination interventions of “cash and care”, in the form of grants combined with parenting. Cash and care provisions together have been shown to reduce transactional and age disparate sex 47. This study shows that “classroom” intervention, in the form of free schooling, may be an additional means to reduce internalizing and externalizing mental health distress and thereby interrupt pathways to HIV‐risk behaviours among adolescent girls in South Africa.

## Competing interests

The authors declare that they have no competing interests.

## Authors’ contributions

FM and MO conceptualized the analysis and the paper and also led the analyses. LC, FM and MO conceptualized the overall study and oversaw data collection. LC contributed to the analyses and interpretation of the findings. All authors reviewed and approved the final version.

## Supporting information


**Table S1.** Proportions of adolescent girls (n = 1498) engaging in HIV risk behaviour based on their risk exposure
**Table S2.** Proportions and mean differences of adolescent girls (n = 1498) exposed to risk based on receipt of free schooling
**Table S3.** Standardized factor loadings for adverse childhood experiences, internalizing and externalizing mental health distress and sexual risk behaviour among South African adolescent girls using simultaneous confirmatory factor analysis (n = 1498)
**Table S4.** Modification steps of the model to achieve final model fit
**Table S5.** Interaction effects of free schooling social protection on pathways from adverse childhood experiences to HIV risk behaviour among adolescent girls (n = 1498)
