# Supplementary material for: Does free schooling affect pathways from adverse childhood experiences via mental health distress to HIV risk among adolescent girls in South Africa: a longitudinal moderated pathway model
Source: J Int AIDS Soc. 2019 Mar 14;22(3):e25262. doi: 10.1002/jia2.25262 (PMC6416665; doi:10.1002/jia2.25262)
Supplement: Supplementary file 1 — Table S1. Proportions of adolescent girls (n = 1498) engaging in HIV risk behaviour based on their risk exposure Table S2. Proportions and mean differences of adolescent girls (n = 1498) exposed to risk based on receipt of free schooling Table S3. Standardized factor loadings for adverse childhood experiences, internalizing and externalizing mental health distress and sexual risk behaviour among South African adolescent girls using simultaneous confirmatory factor analysis (n = 1498) Table S4. Modification steps of the model to achieve final model fit Table S5. Interaction effects of free schooling social protection on pathways from adverse childhood experiences to HIV risk behaviour among adolescent girls (n = 1498) [file JIA2-22-e25262-s001.docx]

Supplementary Table 1: Proportions of adolescent girls (n= 1498) engaging in HIV risk behavior based on their risk exposure

|  | Sex drunk or on drugs | | Inconsistent condom use | | Multiple sex partners | |
| --- | --- | --- | --- | --- | --- | --- |
|  | YES % (n) | NO % (n) | YES % (n) | NO % (n) | YES % (n) | NO % (n) |
| AIDS-ill caregiver | 2.7 (14) | 97.3 (500) | 13.0 (67) | 87.0 (447) | 10.1 (52) | 89.9 (462) |
| Any Abuse | 3.3 (27) | 96.7 (785) | 13.4 (109) | 86.6 (703) | 9.0 (73) | 91.0 (739) |
| Domestic violence | 6.1 (6) | 93.9 (93) | 13.1 (13) | 86.9 (86) | 11.1 (11) | 88.9 (88) |
| Alcohol and drug use | 12.7 (13) | 87.3 (89) | 36.3 (37) | 63.7 (65) | 29.4 (30) | 70.6 (72) |
| Free schooling | 2.0 (21) | 98.0 (1043) | 8.3 (88) | 91.7 (976) | 6.1 (65) | 93.9 (999) |

Supplementary Table 2: Proportions and mean differences of adolescent girls (n=1498) exposed to risk based on receipt of free schooling

|  | Free schooling | |
| --- | --- | --- |
|  | **YES % (n)** | **NO % (n)** |
| AIDS-ill caregiver | 71.0 (365) | 29.0 (149) |
| Non-AIDS-ill caregiver | 71.0 (699) | 29.0 (285) |
| Alcohol and drug use | 66.7 (68) | 71.3% (996) |
| No alcohol and drug use | 33.3 (34) | 28.7 (400) |
| Any Abuse | 69.5 (564) | 30.5 (248) |
| No Abuse | 72.9 (500) | 27.1 (186) |
| Domestic violence | 78.8 (78) | 21.2 (21) |
| No domestic violence | 70.5 (986) | 29.5 (413) |
|  | **YES Mean (SE) SD** | **NO Mean (SE) SD** |
| Anxiety | 5.08 (SE 3.50) SD .107 | 4.89 (SE .18) SD 3.68 |
| Depression | 2.24 (SE .08) SD 2.64 | 1.98 (SE .14) SD 2.94 |
| Suicidal ideation | .42* (SE .98) SD .03 | .67 (SE 1.29) SD .06 |
| Delinquency | 1.69 (SE .06) SD 2.09 | 2.29 (SE .10) SD 2.12 |
| Peer Problems | 4.26 (SE .10) SD 3.35 | 3.93 (SE .15) SD 3.17 |
| Poverty | 1.0 (SE .05) SD 1.59 | .80 (SE .08) SD 1.59 |

* denotes statistically significant differences at p<.05 between the groups using Person’s *χ^2^*-test for binary and independent samples t-test for scale variables

Supplementary Table 3: Standardized factor loadings for adverse childhood experiences, internalizing and externalizing mental health distress and sexual risk behavior among South African adolescent girls using simultaneous confirmatory factor analysis (n= 1498)

| Indicator variable | Adverse experiences | Internalizing | Externalizing | Sexual Risk |
| --- | --- | --- | --- | --- |
| AIDS-ill caregiver | .426*** |  |  |  |
| Child abuse | .469*** |  |  |  |
| Poverty | .430*** |  |  |  |
| Suicide ideation |  | .644*** |  |  |
| Anxiety |  | .761*** |  |  |
| Depression |  | .642*** |  |  |
| Peer problems |  |  | .301*** |  |
| Delinquency |  |  | .320*** |  |
| Drug and alcohol use |  |  | .371*** |  |
| Inconsistent condom use |  |  |  | .855*** |
| Multiple partners |  |  |  | .811*** |
| Sex drunk or on drugs |  |  |  | .724*** |

Note: **** p<.001, model fit: χ^2^/df=4.65, CFI .916, WRMR 1.428, RMSEA .049

Supplementary Table 4: Modification steps of the model to achieve final model fit

| Step | Modification in MPLUS | Explanation |
| --- | --- | --- |
| 1 | !INT ON HIVBL URU | Removal of non-significant path of the control variables baseline HIV risk behavior and urban/rural location on internalizing mental health distress |
| 2 | !HIV ON URU | Removal of non-significant path of the control variable urban/rural location on HIV risk behavior |
| 3 | !ACE ON URU | Removal of non-significant path of control variable urban/rural location on adverse childhood experiences |
| 4 | !HIV ON ACE | Removal of non-significant pathway from adverse childhood experiences to HIV risk behavior |
| 5 | !HIV ON INT | Removal of non-significant pathway from internalizing mental health distress to HIV risk behavior |
| 6 | ALCDR WITH CBCL | Modification based on modification indices – introduction of covariation of error terms of the indicator variables alcohol and drug use and delinquency in the externalizing mental health distress construct |
| 7 | CDI WITH SUIC | Modification based on modification indices – introduction of covariation of error terms of the indicator variables depression and suicidal ideation in the internalizing mental health distress construct |

Supplementary Table 5: Interaction effects of free schooling social protection on pathways from adverse childhood experiences to HIV risk behavior among adolescent girls (n=1498)

|  | Model 1: with interaction of free schooling social protection and ACE | | | Model 2: with interaction of free schooling social protection and internalizing mental health distress | | | Model 3: with interaction of free schooling social protection and externalizing mental health distress | | |
| --- | --- | --- | --- | --- | --- | --- | --- | --- | --- |
| *Predictors* | Internalizing | Externalizing | Sexual Risk | Internalizing | Externalizing | Sexual risk | Internalizing | Externalizing | Sexual risk |
| ACEs | .798*** | .234*** | ns | .686*** | .256*** | ns | .686*** | .262*** | Ns |
| Internalizing | -- | .149* | ns | -- | .128 | ns | -- | .138 | ns |
| Externalizing | -- | -- | .238*** | -- | -- | .326*** | -- | -- | .357*** |
| Free schooling social protection | .032 | -.101* | -- | -- | -.100* | -- | -- | -- | -.079 |
| ACE*Free schooling | -.106* | .010 | -- | -- | -- | -- | -- | -- | -- |
| Internalizing* Free schooling | -- | -- | -- | -- | .007 | -- | -- | -- | -- |
| Externalizing* Free schooling | -- | -- | -- | -- | -- | -- | -- | -- | -.028 |
| Age | .134*** | .074* | .444*** | .129*** | .074* | .443*** | .129*** | .088* | .430*** |
| Baseline sexual risk | .069 | .179*** | .183*** | .074 | .180*** | .184*** | .074 | .201*** | .173*** |
| Rural location | ns | .098* | ns | ns | .099* | ns | ns | .064* | ns |
| *Goodness of Fit* | BIC: 49006.845; AIC: 48725.315 | | | BIC: 49004.158; AIC: 48733.252 | | | BIC: 49007.953; AIC: 48737.047 | | |

Note: all effects reported are standardized, *** p<.001, ** p<.005, * p<.05

-- refers to non-applicable pathways, ns refers to non-significant pathways that were removed in the mediation model
